# The Long-Term Outcomes of Intensive Combined Therapy of Adult Patients with Localised Synovial Sarcoma

**DOI:** 10.3390/jcm9103129

**Published:** 2020-09-28

**Authors:** Katarzyna Kozak, Paweł Teterycz, Tomasz Świtaj, Hanna Koseła-Paterczyk, Sławomir Falkowski, Tadeusz Morysiński, Ewa Bartnik, Anna M. Czarnecka, Michał Wągrodzki, Iwona Ługowska, Piotr Rutkowski

**Affiliations:** 1 Department of Soft Tissue/Bone Sarcoma and Melanoma, Maria Sklodowska-Curie National Research Institute of Oncology, 02-781 Warsaw, Poland; pawel.teterycz@pib-nio.pl (P.T.); tomasz.switaj@pib-nio.pl (T.Ś.); hanna.kosela-paterczyk@pib-nio.pl (H.K.-P.); sfalkowski@coi.waw.pl (S.F.); tmorysinski@coi.waw.pl (T.M.); anna.czarnecka@gmail.com (A.M.C.); iwona.lugowska@pib-nio.pl (I.Ł.); piotr.rutkowski@pib-nio.pl (P.R.); 2 Institute of Genetics and Biotechnology, Faculty of Biology, University of Warsaw, 02-106 Warsaw, Poland; ewambartnik@gmail.com; 3 Institute of Biochemistry and Biophysics, Polish Academy of Sciences, 02-106 Warsaw, Poland; 4 Department of Experimental Pharmacology, Mossakowski Medical Research Centre, Polish Academy of Sciences, 02-106 Warsaw, Poland; 5 Department of Pathology and Laboratory Medicine, Maria Sklodowska-Curie National Research Institute of Oncology, 02-781 Warsaw, Poland; michal.wagrodzki@pib-nio.pl; 6 Early Phase Clinical Trials Unit, Maria Sklodowska-Curie National Research Institute of Oncology, 02-781 Warsaw, Poland

**Keywords:** synovial sarcoma, neoadjuvant, adjuvant, chemotherapy, radiotherapy, prognostic factors

## Abstract

Introduction: Synovial sarcoma (SaSy) is a high-grade, malignant soft tissue sarcoma (STS) accounting for 5–9% of STS. The aim of this study was to analyse outcomes of patients with localised SaSy treated in a single institution with a uniform neo- and adjuvant-combined therapy protocol. Methods: 171 patients with stage II/III SaSy were treated between 1997 and 2014. Chemotherapy consisted of 4 cycles of ifosfamide 12 g/m^2^ and two cycles of a doxorubicin-based regimen 75 mg/m^2^. With the exception of patients who underwent amputation, all patients received neoadjuvant radiotherapy. Results: Median age was 33 years (range 17–69). Tumours larger than 5 cm in size were found in 70% of patients. The 5-year overall survival (OS), local relapse-free survival (LRFS) and metastasis-free survival (MFS) rates were 75%, 80% and 60%, respectively. In multivariate Cox’s regression, age > 35 years, male sex, larger tumour size and histology other than monophasic were associated with worse OS. Conclusions: In adult patients with localised SaSy, long-term survival can be achieved in a significant proportion of cases with intensive combined therapy. The multivariate analysis identified age, sex, disease stage and histology subtype as independent prognostic factors of OS.

## 1. Introduction

Synovial sarcoma (SaSy) is a high-grade malignant mesenchymal tumour which accounts for 5–9% of all soft tissue sarcomas (STS) [1,2,3,4]. SaSy typically affects adolescents and young adults [5,6,7]. There are three main histologic SaSy subtypes: monophasic, biphasic and poorly differentiated. In over 95% of cases of SaSy, the specific chromosomal translocation t(X; 18) (p11.2; q112) is present [8,9,10]. In most cases, this translocation results in rearrangements of the *SYT* gene with the *SSX1* or *SSX2* genes [8]. The most common primary tumour site is the lower limb [4,7,11]. Metastatic lesions at the initial diagnosis are present in approximately 18% of patients [12]. Prognostic factors in patients with SaSy are not well defined. In retrospective analyses, the following are mentioned among factors negatively affecting the prognosis: large primary tumour size, grade 3 tumour, monophasic subtype, male sex, older age at onset, non-extremity location, *SS18–SSX1* fusion and resection R1 [2,5,6,11,13,14,15,16]. The strongest evidence exists for an association between primary tumour size and clinical outcome [2,3,5,7,11,15,17,18]. Wide surgical resection combined with perioperative radiation therapy (RT) is the cornerstone in the treatment of patients with localised SaSy. The role of adjuvant chemotherapy in adult patients with SaSy is not well established. However, according to recently published National Comprehensive Cancer Network (NCCN) and European Society of Medical Oncology (ESMO) guidelines, adjuvant chemotherapy should be considered in high-risk localised STS patients [19,20]. The lung is the most common site of initial recurrence after treatment [4]. Disease recurrence occurs most commonly within 2 years after primary tumour resection, although late relapses after 10 years have been noted [21].

The aim of this study is to evaluate the efficacy, safety and prognostic factors in patients with localised SaSy treated with a uniform neo- and adjuvant-combined therapy protocol.

## 2. Materials and Methods

This prospective study included 171 (96 female, 75 male) patients with locally advanced SaSy treated in the Department of Soft Tissue/Bone Sarcoma and Melanoma at the Maria Sklodowska-Curie National Research Institute of Oncology (MSCNRIO) between 1997 and 2014. The study group comprised adult patients with a primary tumour, after an unplanned tumour excision (≤12 weeks earlier) and with clinical local recurrence. The eighth edition of the American Joint Committee on Cancer (AJCC) classification was used for disease staging. All pathological diagnoses were confirmed in the Department of Pathology, MSCNRIO. In all patients, perioperative chemotherapy was applied according to a uniform scheme following a multidisciplinary evaluation. Preoperative chemotherapy consisted of 2 cycles of ifosfamide at a dose of 1.7 g/m^2^/day on days 1–7 in a cycle of every 21 days. After surgery, patients were administered 2 cycles of doxorubicin, 20 mg/m^2^/day, with cisplatin 35 mg/m^2^/day on days 1–3 in a cycle of every 21 days, and 2 cycles of ifosfamide with the same regimen as in the preoperative setting. Excepting patients who underwent limb amputation, patients received preoperative 5 × 4 Gray (Gy), later modified (from 2005) to 5 × 5 Gy irradiation. One patient with a retroperitoneal tumour received 50.4 Gy in 1.8 Gy per fraction. Radiation therapy RT was administered after 2 cycles of ifosfamide and surgery was performed within 3–5 days after completing RT. In patients, after an earlier unplanned tumour resection, scar re-excision was performed. Patients were followed at 3-month intervals for 2 years, then at 6-month intervals for 3 years and annually thereafter. As the Common Terminology Criteria for Adverse Events (CTCAE) versions have significantly evolved, the tolerance of adjuvant chemotherapy was evaluated according to the CTCAE version 4.0 (Protocol Amendement 01/2010) [22]. The frequency of complications related to wound healing was also evaluated. Ethical approval for this study was obtained from the ethics committee of Maria Sklodowska-Curie National Research Institute of Oncology on 31 March 2009, code number 34/2009. All participating patients signed an informed consent form.

### Statistical Analysis

Statistical analysis was performed in the language and environment R (version 3.5.1), using the tidyverse and survminer packages [23,24]. Descriptive analysis was performed by giving the range of values for numerical variables and the percentage distribution for category variables. Survival curves, median survival with the confidence interval and 5- and 10-year survivals were estimated by the Kaplan–Meier method. The comparison of survival curves, particularly patient subgroups (univariate analysis), was performed using the log-rank test. Overall survival (OS) was calculated from the date of surgery in the frame of combined therapy to the date of death or the date of the last observation in living patients (censored observations). The starting date was the same for the calculations of local relapse-free survival (LRFS), metastasis-free survival (MFS) and disease-free survival (DFS). The final date (complete observations) for LRFS was the date of the local recurrence, for MFS, the date of finding distant metastases, and for DFS, the date of the first recurrence of the disease after finishing combined treatment. In patients in whom disease recurrence was not observed, the final date was the date of the last observation of the patient (censored observations). The Cox regression proportional hazard model was used to evaluate independent factors affecting the patients’ survival. The model encompassed statistically significant variables in univariate analyses as well as the variables which, according to the literature, could affect the patients’ prognoses. The statistical significance level was taken as *p* ≤ 0.05.

## 3. Results

### 3.1. Patients and Treatment

Patient demographics, tumour characteristics and treatment are summarised in Table 1. The median follow-up was 114 months (range, 3–244 months). The median age of patients was 33 years, with a range of 17–69. The most common location was lower limb (*n* = 121), followed by upper limb (*n* = 32). Tumours larger than 5 cm in size were found in 70% of patients (median = 8 cm). The monophasic subtype was more frequent than the biphasic (59% vs. 33%). According to the eighth AJCC staging system, 35 (30%) patients had stage II disease, 55 (47%) patients had stage IIIA disease and 28 (24%) patients had stage IIIB disease. Most patients (*n* = 149, 87%) received all planned chemotherapy cycles, and most patients received 5 × 4 Gy (*n* = 84, 55%) or 5 × 5 Gy (*n* = 69, 43%) irradiation. The median time from initiating preoperative chemotherapy to the surgery itself was 7 weeks. In the group of patients with SaSy localised in the extremities, limb-sparing surgery was performed in 136 patients (89%). Negative surgical margins were reported in 149 of 171 (87%) patients. Adjuvant chemotherapy was discontinued earlier in 11% of patients. The most frequent reason for discontinuation was the occurrence of adverse events (AEs).

### 3.2. Local and Distant Recurrence

Local recurrence occurred in 36 patients (21%). Median time to local recurrence was 26 months (range, 5–137). The 5- and 10-year LRFS was 80% (95% confidence interval (CI) 0.74–0.87) and 74% (95% CI 0.66–0.832), respectively. The 5-year LRFS was 93% in previously untreated patients, 73% in patients with a resection without a prior diagnostic biopsy and 66% in patients treated because of a clinical local recurrence. The incidence of local recurrence in patients referred to the Department prior to any surgical treatment was only 8%. Univariate analysis revealed that male sex (*p* = 0.02), R1 resection (*p* = 0.048) and excision of the primary tumour without a prior biopsy (*p* <0.001) were negative prognostic factors for LRFS (Table 2). In multivariate analysis, only male sex was found to be a statistically significant unfavourable factor for LRFS (HR = 2.81, *p* = 0.04).

Distant relapse developed in 76 patients (44%). Median time to distant relapse was 15.5 months. The lungs were the most common site of metastasis (74%), followed by the lymph nodes (13%). The 5- and 10-year MFS were 60% (95% CI 0.53–0.69) and 51% (95% CI 0.43–0.60), respectively. Univariate analysis identified age > 35 years (*p* = 0.005), male sex (*p* = 0.007), T3/T4 stage (*p* < 0.001) and stage III (*p* < 0.0001) as negative prognostic factors for MFS. In multivariate analysis, the statistically significant prognostic factors associated with shorter MFS were age > 35 years (hazard ratio (HR) = 2.53, *p* = 0.001), histopathological subtype other than monophasic (HR = 1.95, *p* = 0.021) and tumour size as a continuous variable (HR = 1.1, *p* < 0.001) (Table 3). Since the radiation therapy dosing was modified, we separately analysed the outcome of patients treated in 1997–2004 and 2005–2014 periods. However, there was no statistically significant difference in the LRFS (*p* = 0.312) and MFS (*p* = 0.666) between the two groups.

### 3.3. Disease-Free Survival

**Figure 1:**
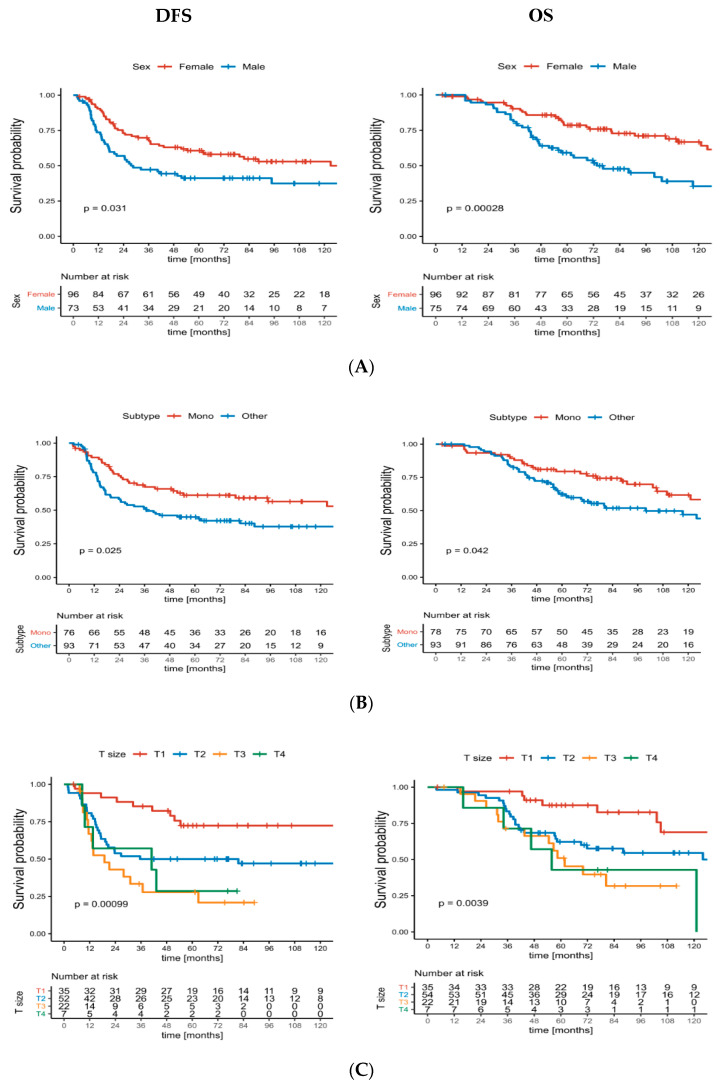

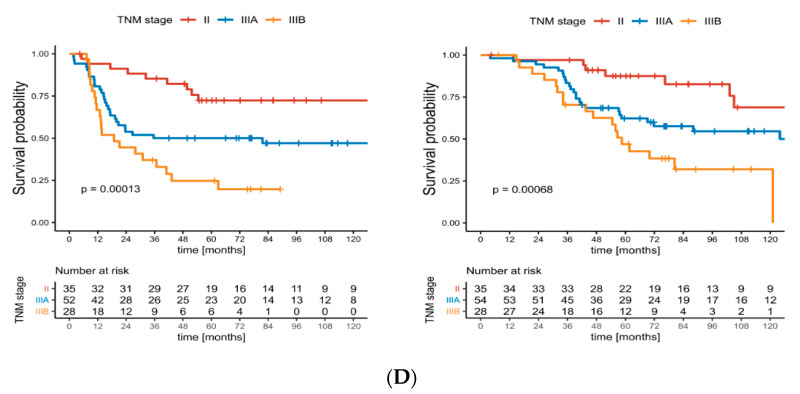
The curves of disease-free survival (DFS) and overall survival (OS) according to (**A**) sex, (**B**) histological subtype, (**C**) T stage and (**D**) TNM (tumour, node, metastasis) stage in patients with localised synovial sarcoma.

The median disease-free survival was 82 months. The 5- and 10-year DFS were 53% (95% CI 0.46–0.62) and 47% (95% CI (0.40–0.56), respectively (Figure 1). Univariate analysis identified the following negative prognostic factors for DFS: age > 35 years (*p* = 0.005), male sex (*p* = 0.03), T3/T4 stage (*p* < 0.001) and stage III (*p* < 0.001). In multivariate analysis, the statistically significant prognostic factors associated with shorter DFS age were: >35 years (HR = 2.39, *p* = 0.001), histopathological subtype other than monophasic (HR = 1.97, *p* = 0.013) and tumour size as a continuous variable (HR = 1.09, *p* < 0.001) (Table 3).

### 3.4. Overall Survival

At the final follow-up, 102 patients (60%) were alive. The 5- and 10-year OS were 75% (95% CI 0.68–0.82) and 58% (95% CI 0.51–0.67), respectively. The impacts of sex, histological subtype, T and TNM stage OS and DFS are illustrated in Figure 1. Univariate analysis revealed that male sex (*p* < 0.001), age > 35 years (*p* = 0.014), T3/T4 stage (*p* = 0.004) and stage III (*p* < 0.001) negatively affected OS. Following multivariate analysis, male sex (HR = 2.18, *p* = 0.006), age > 35 years (HR = 2.03, *p* = 0.012), a histopathological variant other than monophasic (HR = 1.94, *p* = 0.025) and tumour size as a continuous variable (HR = 1.09, *p* < 0.001) were significantly associated with a poor OS (Table 3).

### 3.5. Safety

Treatment was completed in 149 of 171 patients (87%). The most common reasons for adjuvant chemotherapy discontinuation were wound-healing complications (*n* = 8, 5%), haematological toxicity (*n* = 4, 2%), hepatotoxicity (*n* = 2, 1%) and disease progression (*n* = 2, 1%). Thirty patients (18%) had chemotherapy dose reductions. The most common AEs leading to dose reductions were haematological toxicity (*n* = 9, 5%), neurotoxicity (*n* = 8, 5%) and vomiting (*n* = 7, 4%). Common hematologic AEs graded 3 or higher included neutropenia (*n* = 42, 25%) and anaemia (*n* = 11, 6%). However, neutropenic fever was reported in only two (1%) patients. The most frequent non-haematological AEs were nausea (*n* = 61, 36%), vomiting (*n* = 37, 22%) and fatigue (*n* = 35, 20%). Postoperative complications included surgical site infection (*n* = 43, 25%) and wound dehiscence (*n* = 26, 15%). No deaths due to toxicity were reported.

## 4. Discussion

STS are a heterogeneous group of tumours with various histological subtypes characterised by different biologic pathways and various treatment sensitivities. SaSy is considered a relatively chemo-sensitive subtype of STS. A review of 15 European Organisation for Research and Treatment of Cancer (EORTC) advanced first-line STS trials concluded that SaSy patients (*n* = 313) had better progression-free survival (median 6.3 vs. 3.7 months), improved OS (median 15 vs. 11.7 months) and a higher response rate (27.8% vs. 18.8%) compared to STS patients. The overall response rate in patients with SaSy was 21.5% for anthracyclines alone, 32.2% for doxorubicin in combination with ifosfamide and 33% for ifosfamide in monotherapy [25]. However, the role of chemotherapy in the adjuvant treatment of SaSy remains unclear. All studies addressing the role of adjuvant treatment in SaSy patients are retrospective analyses and have conflicting results [2,3,11,17,26,27]. In two large retrospective studies, no benefit of adjuvant chemotherapy (anthracycline, anthracycline + ifosfamide) was observed [2,3]. In contrast, a single institution analysis published by Chen et al. showed that adjuvant chemotherapy (doxorubicin + ifosfamide or doxorubicin + ifosfamide + dacarbazine) in patients with localised extremity SaSy was associated with a significantly better disease-specific survival (DSS) and MFS in patients with stage IIB and III disease (7th AJCC) [17]. In addition, Vining et al., in an analysis of the data derived from the American National Cancer Database (NCDB), observed that adjuvant chemotherapy was associated with better OS in patients with stage III disease (7th AJCC). The impact of adjuvant chemotherapy on OS in this group of patients was observed both in the univariate (HR 0.56; 95% CI 0.33–0.93) as well as the multivariate (HR 0.56; 95% CI 0.33–0.95) analysis [27]. Fewer studies have assessed the role of adjuvant radiotherapy in SaSy patients. Studies from both Surveillance, Epidemiology and End Results (SEER) and NCDB found an overall survival benefit of RT in patients with SaSy undergoing surgery [28,29]. However, two retrospective, single-institution studies demonstrated a significant increase in LRFS with adjuvant RT but no improvement in OS [15,30].

This study was not intended to define the role of adjuvant chemotherapy and radiation therapy in SaSy, because all patients received uniform neo- and adjuvant-combined treatment. Perioperative chemotherapy consisted of four cycles of ifosfamide and two cycles of doxorubicin with cisplatin. In the early 1990s, studies in nonmetastatic STS patients showed high pathologic response rates in patients treated with ifosfamide, doxorubicin, cisplatin and irradiation [31]. Our chemotherapy regimen has been developed on the basis of these encouraging results. Currently, doxorubicin with ifosfamide is considered a standard adjuvant regimen in selected high-risk STS patients [19,20]. The activity of cisplatin monotherapy in metastatic STS patients has been shown to be minimal but Jelic et al. demonstrated that it can act synergistically with epirubicin [32,33]. Based on the results presented above, we cannot conclude that adding cisplatin had any impact on treatment outcomes.

Nevertheless, the present study indicates that long-term survival can be achieved in this group of patients with high-risk tumours. The 5-year LRFS, MFS and OS were 80%, 60% and 75%, respectively. It is important to note that combined treatment had a favourable safety profile, and the low toxicity of preoperative treatment did not delay surgical resection. Most patients (87%) received all planned cycles of chemotherapy. The most common reasons for adjuvant treatment discontinuation were wound-healing complications (*n* = 8, 5%) and haematological toxicity (*n* = 4, 2%). Neutropenia at grade 3–4 was noted in 25% of patients, and neutropenic fever in 1% of patients. The results of the largest analyses concerning treatment of patients with localised SaSy published in the last 20 years are presented in Table 4. For comparison, the results of the present analysis, which is the only one to include a uniform scheme of treatment, are included.

In the literature, a younger age is presented as a favourable prognostic factor for OS in patients with STS [34]. In this study, only patients ≥ 17 years old were enrolled. The multivariate analysis showed that age > 35 years was an unfavourable prognostic factor for OS, MFS and DFS. The results also showed a significant correlation between male sex and worse OS. A similar finding has already been reported by Trassard et al. [7].

The prognostic value of histologic subtype in patients with SaSy remains unclear. Most authors associate the worst prognosis with the poorly differentiated subtype, but because of its very rare incidence, it is difficult to demonstrate a statistically significant difference in comparison to the other subtypes [35]. The prognostic significance of the other subtypes has been evaluated in many studies. In most reports, a tendency to better survival was observed in patients with the biphasic subtype in comparison with the monophasic [2,7,13,16,35]. Moreover, Vining et al. showed that the biphasic subtype was an independent negative prognostic factor for OS (HR 0.41; 95% CI 0.20–0.83) [27]. In contrast to the reports cited above, the present study’s results indicate better outcomes in patients with the monophasic subtype. In the population of patients analysed, the histological subtype was determined in 133/171 patients. The frequency of distribution of particular variants was consistent with the literature data [2,5,26,27]. In univariate analyses, the monophasic subtype in comparison to other subtypes was associated with better OS, MFS and DFS. The prognostic value of this finding was confirmed through multivariate analysis. The 5-year OS in patients with monophasic, biphasic and poorly differentiated subtypes were 79%, 60% and 54%, respectively. These results are intriguing, as they show a statistically significant correlation not described previously in the literature. It can therefore be assumed that this observation may be associated with the effect of adjuvant chemotherapy in some of the patients with SaSy.

Primary tumour size is one of the most important prognostic factors in patients with STS. In the present study, the worst outcome was observed in patients with T3 and T4 stage. The 5-year OS was 51% for T3 stage and 43% for T4 stage. In multivariate analyses, the primary tumour size taken as a continuous variable was a prognostic factor for OS, MFS and DFS but not for LRFS. The impact of the TNM stage on survival in SaSy patients has only been evaluated in several retrospective analyses [6,7]. In the present study, the disease stage was evaluated according to the 8th edition of AJCC [36], which is the first analysis to consider the most recent AJCC classification. In univariate analyses, the TNM stage was a prognostic factor for OS, MFS and DFS. The 5-year OS in stage II, IIIA and IIIB were 88%, 62% and 47%, respectively. These data indicate that the new AJCC classification clearly differentiates patients with stage IIIA from patients with stage IIIB disease. Poor outcomes in patients with stage IIIB disease underline the need for more intensive treatment in this group of patients or to include them in prospective clinical trials with new therapies.

The role of well-planned surgical resection of the tumour in patients with primary STS is indisputable. One of the largest studies evaluating the importance of proper surgical treatment in patients with STS (*n* = 375) showed that in patients directed to a reference centre after prior surgical treatment, more repeated surgeries were necessary and a higher percentage of local recurrences were observed in comparison with patients treated from the beginning in a reference centre [37]. In the present study, univariate analysis demonstrated that planned surgical treatment was a favourable prognostic factor for LRFS. The 5-year LRFS was significantly better in previously untreated patients than in patients with a resection without a prior diagnostic biopsy (93% vs. 73%, respectively). The prognostic significance of resection margins in patients with SaSy has been evaluated in numerous retrospective analyses. In univariate analyses, an unfavourable effect of positive margins has been observed on LRFS [6], DSS [7,16] and MFS [5,7]. Moreover, Italiano et al. showed that R1 resection adversely affected OS and LRFS in multivariate analysis [3]. In the present study, local relapse rate was nearly two times higher after R1 resection when compared to R0 resection. The 5-year LRFS in patients after R0 resection in comparison with patients after a microscopically non-radical resection was 82% and 69% (*p* = 0.048), respectively. Importantly, the incidence of local recurrence in patients referred to the Department prior to any surgical treatment was only 8%. These findings support the referral of patients with a suspected STS to specialised centres prior to any treatment.

## 5. Conclusions

In conclusion, the results of this study demonstrate long-term survival in adult patients with localised SaSy treated with neo- and adjuvant-combined treatment. The key strengths of this study are the large number of patients included, the uniform mode of treatment and the long period of follow-up. Of note are the positive treatment results obtained despite the high percentage of patients with a primary tumour size larger than 5 cm. These results confirm the importance of planned surgery and clear surgical margins for local control. They suggest that age, sex, disease stage and histological subtype are independent prognostic factors of OS.

## Figures and Tables

**Table 1:** Characteristics of patients with locally advanced synovial sarcoma (*n* = 171).

Characteristic	*n*	%
**Age**		
≤35	102	60
>35	69	40
**Sex**		
Female	96	56
Male	75	44
**Histological subtype ^a^**		
Monophasic	78	59
Biphasic	44	33
Poorly differentiated	11	8
**Localisation of primary tumour**		
Upper limb	32	19
Lower limb	121	71
Trunk wall	15	9
Retroperitoneal space and head and neck	3	2
**Size of primary tumour, cm ^b^**		
≤5	35	30
>5	83	70
**Disease stage according to AJCC ^b^**		
II	35	30
IIIA	55	47
IIIB	28	24
**Status at initiation of treatment in MSCNRIO**		
Patients previously untreated (primary tumour)	77	45
Patients after surgical treatment without prior diagnostic biopsy	64	37
Clinical local recurrence	30	18
**Type of surgical treatment for localisation in an extremity**		
Limb-sparing surgery	136	89
Amputation	17	11
**Surgical margins**		
R0	149	87
R1	22	13
**Preoperative radiotherapy**	154	
5 × 4 Gy	84	55
5 × 5 Gy	69	45
28 × 1.8 Gy	1	1
**Number of cycles of perioperative adjuvant chemotherapy**		
six	149	87
five	6	4
four	7	4
three	6	4
two	3	2

^a^—In some patients, the data were not included in the histopathological report. ^b^—In some patients, the data concerning the primary tumour resected outside the MSCNRIO were not available. MSCRNIO—Maria Sklodowska-Curie National Research Institute of Oncology, AJCC—American Joint Committee on Cancer. Gy—Gray.

**Table 2:** Univariate analysis of variable factors for LRFS, MFS and OS.

Variable	5-Year LRFS	95% CI	*p*	5-Year MFS	95% CI	*p*	5-Year OS	95% CI	*p*
**Age**									
≤35	80%	0.72–0.89	0.82	66%	0.58–0.76	0.0051	81%	0.73–0.88	0.014
>35	81%	0.70–0.93		52%	0.41–0.66		67%	0.57–0.80	
**Sex**									
Female	88%	0.81–0.95	0.02	70%	0.62–0.81	0.0068	84%	0.76–0.92	<0.001
Male	70%	0.59–0.83		48%	0.37–0.61		64%	0.54–0.76	
**Histological subtype**									
Monophasic	78%	0.69–0.89	0.39	74%	0.64–0.49	0.08	79%	0.71–0.89	0.2
Biphasic	84%	0.73–0.96		44%	0.32–0.62		60%	0.47–0.77	
Poorly differentiated	100%	1.00–1.00		50%	0.27–0.92		54%	0.26–1.00	
Not specified	76%	0.63–0.93		58%	0.44–0.76		65%	0.51–0.82	
**T stage**									
T1	84%	0.71–0.98	0.97	85%	0.74–0.98	<0.001	88%	0.77–0.99	0.0039
T2	88%	0.79–0.98		53%	0.41–0.69		62%	0.50–0.77	
T3	85%	0.71–1.00		32%	0.17–0.61		51%	0.33–0.78	
T4	86%	0.63–1.00		29%	0.09–0.92		43%	0.18–1.00	
**TNM stage**									
II	84%	0.71–0.98	0.86	85%	0.74–0.98	<0.001	88%	0.77–0.99	<0.001
IIIA	88%	0.79–0.98		53%	0.41–0.69		62%	0.50–0.77	
IIIB	85%	0.72–1.00		28%	0.15–0.52		47%	0.31–0.71	
**Surgical margins**									
R0	82%	0.76–0.89	0.048	62%	0.55–0.71	0.57	73%	0.66–0.81	0.25
R1	69%	0.51–0.93		50%	0.33–0.76		53%	0.35–0.80	

Abbreviations: CI, confidence interval; LRFS, local recurrence-free survival; MFS, metastatic-free survival; OS, overall survival; TNM (tumour, node, metastasis).

**Table 3:** Multivariate analysis of variable factors for MFS, DFS and OS.

Variables	MFS HR (95% CI), *p*	DFS HR (95% CI), *p*	OS HR (95% CI), *p*
Male sex	1.53 (0.90–2.60), *p* = 0.117	1.60 (0.97–2.64), *p* = 0.067	2.18 (1.25–3.78), *p* = 0.006
Age > 35 years	2.53 (1.48–4.34), *p* = 0.001	2.39 (1.44–3.96), *p* = 0.001	2.03 (1.17–3.52), *p* = 0.012
Histologic subtype other than monophasic	1.95 (1.11–3.44), *p* = 0.021	1.97 (1.15–3.35), *p* = 0.013	1.94 (1.09–3.44), *p* = 0.025
Tumour size (continuous variable)	1.10 (1.05–1.16), *p* < 0.001	1.09 (1.04–1.14), *p* < 0.001	1.09 (1.04–1.14), *p* < 0.001

Abbreviations: CI, confidence interval; DFS, disease-free survival; HR, hazard ratio; MFS, metastatic-free survival; OS, overall survival.

**Table 4:** Comparison of treatment results with the largest analyses concerning treatment of patients with localised synovial sarcoma.

Study	Number of Patients	Size of Primary Tumour (%)	Median Follow-Up Time (Months)	R1 Resection (%)	RT Periop (%)	CHT (%)	5-Year LRFs (%)	5-Year MFS (%)	5-Year OS (%)
Canter et al. (2008) [18]	255	≥5 cm	72	-	63	39	-	55	72
		56							
Chen et al. (2012) [17]	76	≥5 cm	68	32	75	68	-	48	59
		100							
Italiano et al. (2009) [3]	237	-	58	15	76	60	70	57	64
Lewis et al. (2000) [5]	112	≥5 cm	72	14	46	37	78	61	75
		45							
Palmerini et al. (2009) [2]	204	>5 cm	66	12	52	52	81	-	76
		49							
Trassard et al. (2001) [7]	128	≥5 cm	37	24	80	57	-	-	63
		70							
This study (2020)	171	≥5 cm	114	13	90	100	80	60	75
		73							
Ferrari et al. * (2004) [11]	215	>5 cm	65	-	50	28	63	51	71
		64							
Outani et al. * (2019) [15]	191	≥5 cm	68	7	55	30	89	66	76
		63							

* The analyses encompass both adult patients and children. Abbreviations: CHT, chemotherapy; LRFS, local recurrence-free survival; MFS, metastatic-free survival; OS, overall survival; RT periop, perioperative radiation therapy; -, not reported.
